# Supplementary material for: Complexome profiling of the Chlamydomonas psb28 mutant reveals TEF5 as an early PSII assembly factor
Source: Plant Cell. 2025 Mar 17;37(6):koaf055. doi: 10.1093/plcell/koaf055 (PMC12164590; doi:10.1093/plcell/koaf055)
Supplement: koaf055_Supplementary_Data [file koaf055_supplementary_data.zip › psb28_interactive.zip!/plots/intensity/qualityPlots/peptides/_Cre06.g294050.t1.1.html]

### Cre06.g294050.t1.1

Trivial name:   
SeqCoverage [median]: 0.70  
Mass: 288.63 kDa  
  
 

| Sequence | MissedCleavage | Mass [Da] | wtIntensity | muIntensity | Score |
| --- | --- | --- | --- | --- | --- |
| ELALEPGSGR | 0 | 1027.53 | 7683.95 | 13512.84 | 68.92 |
| IIVAGDVVR | 0 | 940.57 | 0.00 | 6030.28 | 65.04 |
|  |  |  | 7684.0 | 19543.1 |  |
